# Anxiety and intellectual functioning in autistic children: A systematic review and meta-analysis

**DOI:** 10.1177/1362361320953253

**Published:** 2020-11-16

**Authors:** Jessica E Mingins, Joanne Tarver, Jane Waite, Chris Jones, Andrew DR Surtees

**Affiliations:** 1 University of Birmingham, UK; 2 Aston University, UK; 3 Birmingham Children’s Hospital, UK

**Keywords:** anxiety, autism, autism spectrum disorder, intelligence quotient, meta-analysis

## Abstract

Autistic children experience higher levels of anxiety than their peers. Making appropriate diagnoses of anxiety disorders and providing effective treatment for these children is particularly difficult. Inconsistent evidence suggests that levels of anxiety in autistic children are related to intellectual functioning. We provide the first meta-analysis of this evidence. A systematic search identified 49 papers for review. These papers included measures of anxiety and intelligence quotient in 18,430 autistic children. Studies employing correlations showed evidence of a significant relationship between intelligence quotient and anxiety in autistic children: children with higher intelligence quotient scored higher on measures of anxiety. Studies directly comparing groups of autistic children with and without intellectual disability also supported this conclusion. Most studies employing other designs also supported this finding. Employing a quality assessment framework identified common threats to validity. Many studies used measures of anxiety that were not validated across the samples they measure. This was most notable for those autistic children with an intellectual disability. It is vital that future research determines whether the identified relationship between intelligence quotient and anxiety reflects something important in the mechanism for anxiety in autistic children, *or* quantifies the lack of sensitivity of our measures of anxiety across different groups.

**Lay abstract:**

Autistic children often experience higher levels of anxiety than their peers. It can be difficult to diagnose and treat anxiety disorders in autistic children, in part because of the high degree of variability in their underlying abilities and presentations. Some evidence suggests that autistic children with higher intelligence (as measured by intelligence quotient) experience higher levels of anxiety than autistic children with lower intelligence. However, the evidence is inconsistent, with other papers not finding a difference or finding higher levels of anxiety in autistic children with lower intelligence. In this article, we review existing literature to see whether autistic children with higher intelligence quotients have higher anxiety than autistic children with lower intelligence quotients. A systematic search of the literature was conducted which identified 49 papers on the topic. The methods of all the papers were reviewed using an objective quality assessment framework. When combining the data statistically, there was evidence that autistic children with higher intelligence quotients are more anxious than autistic children with lower intelligence quotients. The quality review raised common weaknesses across studies. Most importantly, few studies used measures of anxiety that have been shown to be valid for children with very low intelligence quotients. Similarly, many studies used measures of anxiety that have not been shown to be valid for autistic children. These factors are important because autistic children and those with low intelligence quotient may experience or understand anxiety differently. Future research should use fully validated measures to test whether high intelligence quotient is associated with high levels of anxiety in autistic children.

Autism is characterised by difficulties in social communication and interaction across multiple contexts, as well as restricted or repetitive behaviours or interests (American Psychiatric Association, 2013). Approximately 1%–2% of children^1^ in the United Kingdom and the United States are autistic, with rates of diagnosis increasing each year (Baio et al., 2018; Russell et al., 2014). Autism characteristics vary between people, and symptoms often co-occur with other neurodevelopmental or psychiatric disorders (de Bruin et al., 2007; Leyfer et al., 2006). Among the most common comorbidities are anxiety disorders (de Bruin et al., 2007; Lai et al., 2019; Mattila et al., 2010). Anxiety disorders comprise a range of related conditions, characterised by an emotional experience of anxiety, allied with excessive worry (American Psychiatric Association, 2013). Behavioural changes and physical symptoms are common in anxiety disorders, including avoidance, sleeping difficulties and muscle tension (American Psychiatric Association, 2013).

## Anxiety in autism

Around 40% of autistic children meet criteria for an anxiety diagnosis (Mattila et al., 2010; Simonoff et al., 2008), with estimates ranging from 11% to 84% (White et al., 2009), as opposed to 2%–24% in the general population (Merikangas et al., 2009). Autistic children are twice as likely as their neurotypical counterparts to receive an anxiety disorder diagnosis (van Steensel et al., 2011). Anxiety disorder trajectories in autistic children are similar to children with anxiety disorders alone, often presenting as externalising behaviours in younger children, which changes to withdrawal and avoidance in adolescence (American Psychiatric Association, 2013; Kerns & Kendall, 2012; White et al., 2009). However, autistic children experience more compulsions, higher social avoidance and anxieties linked to sensory sensitivities (Acker et al., 2018). Understanding anxiety in autistic children is essential, as having both an anxiety disorder and being autistic is associated with increased self-injurious behaviour, depression and parental stress compared to autism alone (Kerns et al., 2015) and decreased quality of life (van Steensel et al., 2012). There is also substantial evidence that understanding anxiety disorders in autism is a research priority for the autism community (James Lind Alliance, 2016; Pellicano et al., 2014).

## Autism, anxiety and intellectual functioning

Intellectual functioning varies significantly in autistic children (Charman et al., 2011). Around half of autistic children have intelligence quotients (IQs) within the normal range or above, but half have a comorbid intellectual disability (ID; Charman et al., 2011), the diagnostic criteria for which necessitate an IQ < 70. There is evidence that those autistic children with the lowest IQ (those with comorbid ID) show a different phenotype of autism, including increased risk of challenging behaviours and decreased adaptive behaviour (Matson & Shoemaker, 2009). It is therefore important to consider whether intellectual functioning is predictive of other difficulties in autistic children.

There is some evidence suggesting anxiety varies with IQ in autistic children, though contradictory findings exist. High IQ has been associated with anxiety disorders in autistic children (Salazar et al., 2015). Conversely, other studies have suggested that low IQ may be associated with more difficulties with anxiety in autistic children (Rosenberg et al., 2011; van Steensel et al., 2011). A recent meta-analysis (van Steensel & Heeman, 2017) showed that differences in anxiety between autistic children and their peers were greatest for those with highest IQ. This article only looked at studies that made a direct comparison to neurotypical children and only synthesised the comparative data. Furthermore, it excluded papers with children with comorbid ID. The evidence for a relationship between IQ and anxiety in autistic children remains unsynthesised. Research in this area, however, presents significant challenges.

## Challenges with researching anxiety in autism and low IQ groups

High symptom overlap between anxiety disorders and autism means that there are few good measures for anxiety in autistic children (Wood & Gadow, 2010). There is little consistency in how anxiety in autistic children is measured in the literature and a lack of consensus around best practice measures (White et al., 2009). This makes giving a clinical diagnosis difficult and means there is high methodological variability in published studies. Furthermore, the high prevalence of alexithymia – the difficulty to recognise and express emotions – in autistic children (around 55%; Kinnaird et al., 2019) may cause problems with measuring anxiety, although research into this area is limited. The majority of studies of anxiety in autistic children rely on parent report. Relying on parent report may be problematic, as this often requires a child to be able to express their emotions to their caregiver. Despite these difficulties, some well-validated measurement tools for anxiety in autistic children do exist, such as the Child Adolescent Symptom Inventory–Anxiety (CASI-Anx; Sprafkin et al., 2002) and the Spence Children’s Anxiety Scale–Parent Report (SCAS-P; Jitlina et al., 2017).

Additional difficulties with reliability present when we consider those autistic children with ID. Psychiatric disorders such as anxiety are often underdiagnosed in people with ID, as diagnostic criteria and assessment methods of anxiety rely heavily on verbalisation or communication of anxiety through self or parent reports (Bailey & Andrews, 2003; Matson et al., 1997). People with ID often have limited communication ability, and so may not be able to express their worries verbally, or label complex internal states such as anxiety. Despite this, heightened prevalence estimates of anxiety in ID remain (Emerson & Hatton, 2007). Some measures have been validated for measuring anxiety in children with ID, such as the Strengths and Difficulties Questionnaire (SDQ; Emerson, 2005).

Measuring anxiety in autistic children with comorbid ID presents further difficulties, and there are much fewer appropriate measures of anxiety for this population. Measures of anxiety seem to be modified for use in ‘high-functioning’ autism *or* developed for those with ID. Few take into consideration the effects of both on anxiety, and there is no gold standard measure of anxiety for autistic children with comorbid ID. One measure which has adequate reliability and validity in a sample of autistic children with a range of IQs (42–141) is the Autism Comorbidity Interview, Present and Lifetime Version (ACI-PL; Leyfer et al., 2006), but this is not widely used.

## Rationale

Existing literature presents unclear and contradictory estimates of the relationship between IQ and anxiety in autistic children and often ignores the large proportion of children with comorbid ID. In contrast, this meta-analytic review will quantify the current state of the evidence for the relationship between IQ and anxiety in autistic children over a broad range of intellectual abilities. Furthermore, we will estimate whether including children with ID affects estimates, as these children are often underrepresented in anxiety research despite making up 50% of the autistic population (Charman et al., 2011). Existing literature often uses anxiety measures with limited evidence of validity for autistic children. A quality assessment framework (QAF) will be employed to identify the role of methodological variation including measures of anxiety and sample on the results. Having a clear picture of how anxiety presents in autism across a range of IQ scores is vital to understanding who is most at risk and may prevent diagnostic overshadowing. This will allow better access to services, such as cognitive behavioural therapy (CBT), shown to be effective for anxiety in an autistic group when appropriately modified (van Steensel & Bögels, 2015).

## Method

### Search strategy

A systematic search of the literature on IQ and anxiety in autistic children was conducted through PsycINFO (1967–March 2020), PsycARTICLES (1967–March 2020), and MEDLINE (1946–March 2020), using Ovid. Search terms used were (‘autis*’ or ‘Asperger*’ or ‘PDD’ or ‘pervasive developmental adj1 disorder’ or ‘ASD’) AND (‘anxi*’ or ‘anxiety adj1 disorder’) AND (‘IQ’ or ‘intelligence adj1 quotient’ or ‘intelligen*’). These search terms were combined, and duplicates were removed, producing 1252 results. Papers were screened by initial eligibility criteria whereby any papers not in English, not in peer-reviewed journals or using a non-human sample were removed.

### Paper selection

**Figure 1.**
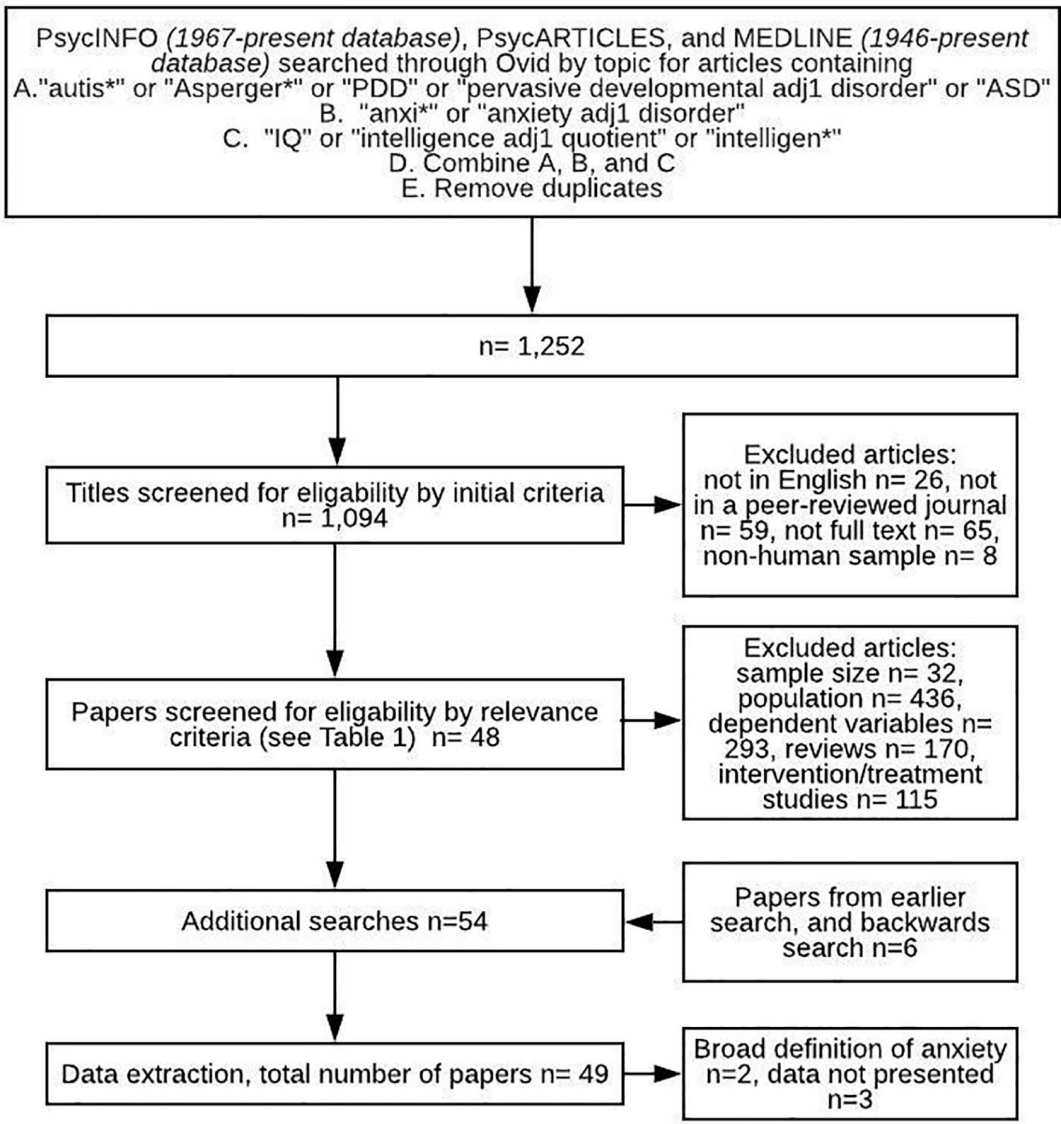
Full search strategy.

The full search strategy, using Preferred Reporting Items for Systematic reviews and Meta-Analyses (PRISMA) guidelines, is described in Figure 1. Titles and abstracts were screened by inclusion and exclusion criteria (Table 1) and removed if they met any exclusion criteria or did not meet all inclusion criteria. If this could not be determined from title and abstract, the full paper was screened. Papers in the backwards literature search were screened in the same way. The search produced 54 eligible papers. A further five papers were excluded at the data extraction stage. This was either because they did not include a specific measure of anxiety, instead reporting ‘internalising symptoms’ (Simonoff et al., 2013; van steensel et al., 2013) or because they did not present sufficient data for extraction (Johnson et al., 2015; Mayes & Calhoun, 2004; Ozsivadjian et al., 2014). Where papers did not present enough data for extraction, authors were contacted to obtain this information, but either declined or did not respond. Therefore, the total number of papers included in this review was 49. To ensure validity of the search, a second researcher independently screened 20% of the total search for eligibility and inclusion, for this 20% of studies, researchers agreed on inclusion in all cases.

**Table 1.** Inclusion and exclusion criteria.

Inclusion criteria	Exclusion criteria
a. Population: Reports data from a population of autistic children and young people. Papers are included on the basis of author report of Autism or an Autism Spectrum Disorder (including Asperger Syndrome or Pervasive Developmental Disorder – Not Otherwise Specified). Mean age of the population must be <18 years, with range <25. b. Sample size: Sample size (*n* ⩾ 5)	a. Reviews: No original data reported) b. Intervention/treatment: Measures of anxiety form part of an intervention or treatment study
c. Dependent variables i. Measures anxiety: Authors report data from a measure of anxiety or anxiety disorder ii. Measures intellectual functioning: Authors report data on intellectual functioning of participants. Clinical diagnosis of intellectual disability alone accepted in absence of reported IQ scores. iii. Data analysis/reporting: Authors report data that can be used to quantify the relationship between measure of anxiety and measure of intellectual functioning (e.g. correlation or means of/t tests between different groups).	

IQ: intelligence quotient.

### Data extraction

Data extracted from each paper included demographic information and information regarding sample and measures including sample size, recruitment process, exclusions based on demographic variables, identification and recruitment of autistic children, measurement of intellectual functioning and measurement of anxiety (see Supplementary Table 1). Where available, scores from parent reports of anxiety were meta-analysed as this was the most common choice of informant when collecting information about anxiety across studies. Given the relatively small size of the literature, decision-making favoured inclusion of a general measure of anxiety. Therefore, where possible, anxiety scores extracted from papers were those which reflected total anxiety as opposed to specific anxiety disorders such as separation anxiety or social phobia. Where papers presented data for multiple anxiety measures (*N* = 2), those with the highest reliability statistics, as presented by the authors, were used. A second researcher independently checked data extracted to ensure data quality.

### Quality review

A QAF (Table 2) was developed, based on Surtees et al. (2018) and Richards et al. (2015). This was designed to measure the methodological limitations of a study in relation to the goals of the meta-analysis, such as problems with sample or measurements of IQ or anxiety. Each paper was rated (0–3) on five factors, with overall score being the mean of these ratings.

**Table 2.** Quality assessment framework.

Item	Poor (0)	Fair (1)	Good (2)	Excellent (3)
Sample **identification of an autistic sample**	Unspecified	Single restricted or non-random sample, for example, a specialist clinic or previous research study Single regional sample, for example, a regional parent support group	Multiple restricted or non-random samples, for example, multi-region specialist clinics, multiple schools National non-random sampling, for example, national parent support groups	Random sample
Measurement **of autism** **Reliability/validity of measurement of autism**	Unspecified	Part of a group known to be at risk of autism (parents or siblings with autism) Self/parent/teacher report Recruitment from specialist school or support group High score on screening questionnaires such as Autism Spectrum Quotient (AQ)	Best-estimate diagnosis by clinician High score on one or more validated measures of autism, for example (ADOS, ADI-R) Previous diagnosis of autism by multidisciplinary team using multiple assessment methods unconfirmed in the present study OR confirmed in the present study using only ONE assessment method or measure	Clinical diagnosis confirmed by a multidisciplinary team using DSM-IV, DSM-V or ICD-10 criteria and multiple, well-validated assessment tools (e.g. ADOS, ADI-R) Diagnosis must be confirmed in this study – not in previous studies or as part of their assessment through a clinic. Diagnosis must also be confirmed using multiple (two or more) assessment methods and a multidisciplinary team (i.e. at least TWO professionals must have come to this verdict, can be speech and language therapist/OT/psychiatrists/psychologists etc.)
Measurement **of IQ** **Reliability/validity of IQ measure**	Unspecified For example, ‘an IQ measure/test’ without later description	Self/parent/teacher report Self/parent/teacher report using a well-validated measure, but not validated AT ALL for the present sample (not validated for low IQ or autistic groups)	Self/parent/teacher report using a well-validated measure – but not FULLY validated for the present population. For example, in a study using ID and comorbid autism, it may be validated in only one of these populations. Previous IQ test not repeated for the study – authors must specify that a formal IQ test was conducted in the past OR mention a specific test	Formal IQ test (e.g. Wechsler Intelligence Scale for Children, Mullen Scales of Early Learning, Stanford-Binet), validated in the present population. Specific IQ test must be specified
Measurement **of anxiety** **Reliability/validity of anxiety measure**	Unspecified	Self/parent/teacher report Self/parent/teacher report using a well-validated measure, but not validated AT ALL for the present sample (not validated for low IQ or autistic groups). (CBCL, RCADS, Paediatric Behaviour Scale, KID-SCID)	Self/parent/teacher report using a well-validated measure – but not FULLY validated for the present population. For example, in a study using ID and comorbid autism, it may be validated in only one of these populations (SDQ, CASI, SCAS-P, BASC). Adaptations of well-validated measures to make them SOMEWHAT appropriate for the present population. For example, in a study using ID and comorbid autism, it may have been adapted only for one of these populations	Formal measure (such as Autism Comorbidity Interview, Present and Lifetime Version ACI-PL) validated in the present population. To score excellent, a measure must be validated in both autism and ID. If a measure has been modified because there is little validation for autistic/ID groups, it must be modified to for BOTH autism and ID, as specified by the authors
Appropriate **use of statistical tests**	Unspecified or inappropriate analysis	NA	NA	Appropriate analysis using statistical tests

IQ: intelligence quotient; DSM-IV: *Diagnostic and Statistical Manual of Mental Disorders* (4th ed.); DSM-V: *Diagnostic and Statistical Manual of Mental Disorders* (5th ed.); ADOS: Autism Diagnostic Observation Schedule; ADI-R: Autism Diagnostic Interview-Revised; ICD-10: International Classification of Diseases, Tenth Revision; CBCL: Child Behaviour Checklist; RCADS: Revised Children’s Anxiety and Depression Scale; KID-SCID: Structured Clinical Interview for DSM-IV Psychiatric Diagnoses; SDQ: Strengths and Difficulties Questionnaire; CASI: Child and Adolescent Symptom Inventory; SCAS-P: Spence Children’s Anxiety Scale–Parent; BASC: Behaviour Assessment System for Children.

Two factors referred to ensuring that the sample was appropriate for the study. The first of these described the process of recruitment, with a random sample being excellent, and samples from one source or group being rated as fair (such as one outpatient unit). The second factor referred to the confirmation of the presence of autism spectrum disorder (ASD) diagnosis. Studies were considered excellent if a clinical diagnosis was confirmed in their study by a multidisciplinary team using a diagnostic manual and multiple tools, as is consistent with National Institute for Health and Care Excellence (NICE) guidelines for ASD diagnosis (NICE, 2011). Studies were given a rating of fair if they were part of an at-risk group, scored highly on screening measures, or were recruited from specialist schools with no confirmation of diagnosis.

Another two factors were designed to measure the reliability and validity of measures used in the studies. These were the measurement of IQ and anxiety, respectively. In terms of IQ, studies were rated as excellent if they used a formal IQ test, validated for use in their population. Well-validated measures, but which had not been validated specifically for use in ASD or ID, and parent reports of clinical diagnosis of ID without reported IQ scores were given a rating of fair. The same criteria were used for measurement of anxiety in that a score of excellent required full validation for their sample (validated in both ASD and ID groups), good required partial validation (validated in only one of ASD or ID) and fair had no validation for their sample (not validated for ASD or ID specifically).

The final factor measured analysis and was scored only as poor or excellent with no intermediate values, in contrast to the other factors. A score of excellent was given if statistical analysis was appropriate, and a score of zero was given if analysis was not specified.

The quality framework was revised following initial inter-rater reliability analysis on a subset of the papers. The final version of the criteria was used by two independent raters to score all papers, and a good degree of inter-rater reliability observed, *k* = 0.91. Any discrepancies were discussed and final scores were agreed upon.

## Results

### Participants and designs

Across the 49 papers returned, there were 18,430 participants. Two main study designs were identified: correlations between IQ and score on an anxiety measure and group-level data analysed through t tests. In all, 31 papers employing correlations were included. These studies form the basis of meta-analysis 1 (*N* = 8267). Eight papers used t tests to compare groups. Four split the data by ID versus non-ID groups and compared anxiety scores between groups. These data form the basis of meta-analysis 2 (*N* = 761). While the number of studies is low, sample sizes were high. Four papers compared mean IQ scores of high versus low anxiety groups. These papers were not meta-analysed as total sample size was considerably lower than papers which split by IQ (*N* = 237). These papers are included in the narrative review, alongside other analytic strategies.

### Study quality

The mean overall quality of papers was 2.1/3 (range 1.0–2.6) on the quality framework (Table 2). Recruitment of an autistic sample was rated as fair to good (mean quality = 1.6/3). Some studies recruited from multiple sources, such as multiple support networks or outpatient centres, although none were able to recruit a truly random sample. Some studies were more limited in that they recruited from a single geographical location or centre, but none failed to specify their recruitment process. Most studies had fair to good means of assessing autism (mean quality = 2.0/3), and studies predominantly reported that a previous clinical diagnosis of autism was essential for inclusion in the study. Most studies scored excellent or good for assessment of IQ (mean quality = 2.2/3), as most used a version of the Wechsler Intelligence Scales (Wechsler, 2014). Most used anxiety measures that were not validated for use in autistic or ID groups (mean quality = 1.6/3). Some studies made attempts to modify their measures to prevent overlap in symptomatology in autism and anxiety but very few made alterations for ID groups. Most studies used parent reports of anxiety. Many measures relied on parental recognition of behavioural symptoms of anxiety, which may be qualitatively different in children with ID, and hence not be detected by measures developed for use in neurotypical children (Rodgers et al., 2012). All studies conducted appropriate analyses and therefore scored excellent (mean quality = 3.0/3) on this factor.

### Meta-analysis

#### Analysis strategy

Data were analysed using R (R Core Team, 2013). Two meta-analyses were conducted: meta-analysis 1 examined the correlational data and meta-analysis 2 the group-level data, split by IQ. For each of these meta-analyses, random and quality effects models (QEMs) were tested. A random effects model (REM) assumes that variability between studies relates to random variation. It therefore weights each study based on the number of participants and the variation from findings across the full set of studies. A QEM assumes that some of the variation between studies is explained by the quality of the methodology employed. A QEM weights contribution to the model by number of participants *and* quality ratings. Given the relatively small literature, our search strategy prioritised an inclusive approach, including all papers in the initial analysis. To investigate the effects of this, we conducted further analysis to confirm whether the results were consistent if papers were included that used a broader age range (<25), in a sample size with mean age <18 (Syriopoulou-Delli et al., 2019).

We performed subgroup analysis to understand whether any observed effect was consistent across studies with a different approach to including children with ID. For this, three groups were created. The *ID Included* group comprises those studies that either explicitly reported including children with ID or reported a range of IQs with a minimum <70. The *No ID* group comprises those studies that either explicitly reported excluding children with ID or included a range of IQs with a minimum ⩾70. The *Unclear* group includes those studies that did not explicitly report including or excluding children with ID and did not report on IQ range. Subgroup analysis allows for determining whether a different pattern of results was observed across these groups *and* for calculating independent estimates of the effect for each of these subgroups.

##### Meta-analysis 1: correlations

**Figure 2.**
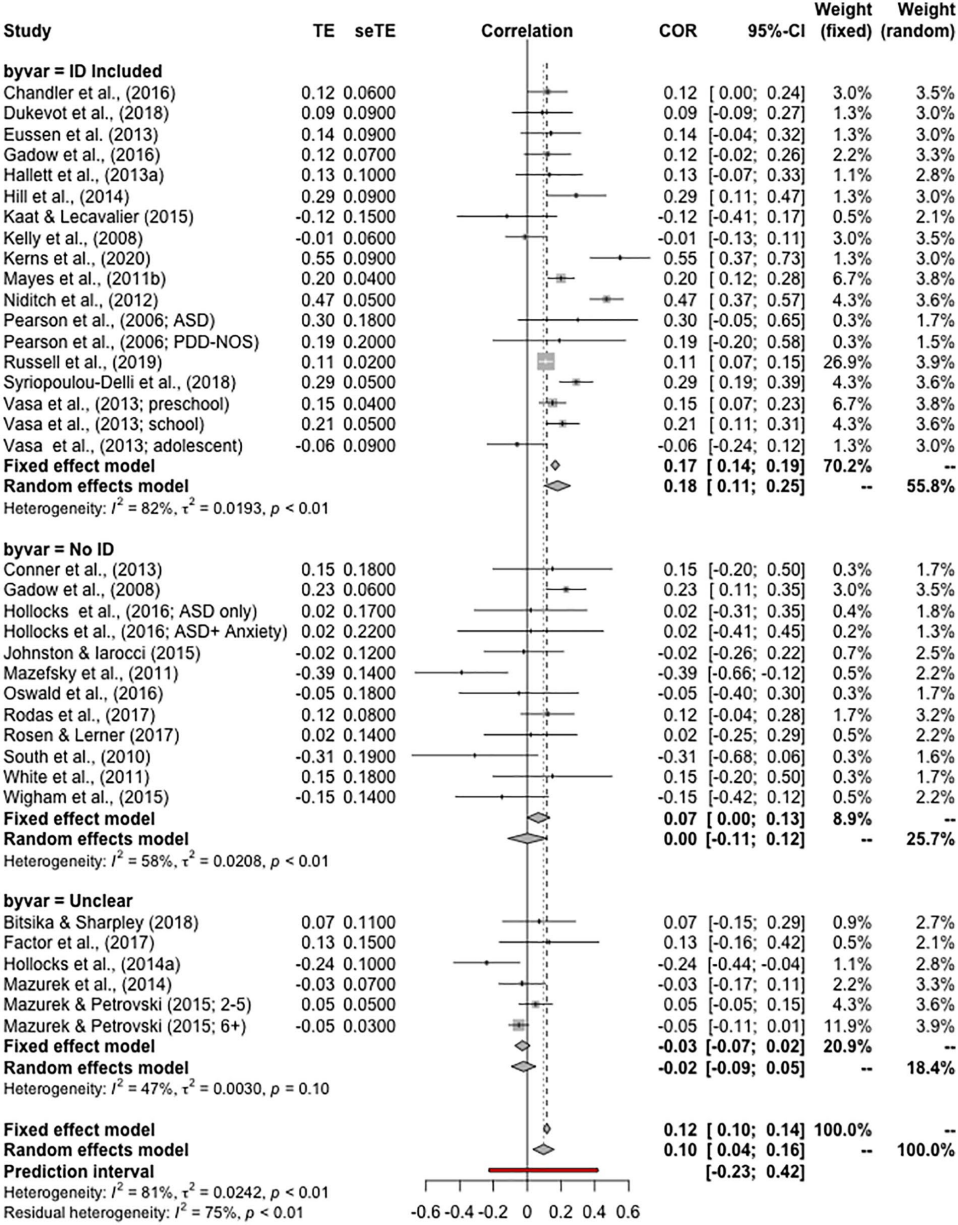
Forest plot of the random effects model of meta-analysis 1 – on correlations between IQ and anxiety in autistic children. Data are split by subgroups (‘byvar’) based on whether studies included participants with intellectual disabilities.

A total of 31 papers included a correlation analysis, with data from 8267 participants. A REM was calculated using the restricted maximum likelihood estimator. This estimator is a more robust version of traditional DerSimonian–Laird estimates in non-normal distributions of effect, as it restricts the likelihood estimates to control for underestimation and minimises bias (Cheung, 2013). Results of the REM indicated that there was a small but significant positive correlation between IQ scores and anxiety scores in autistic children, *r* = 0.10, 95% confidence interval (CI): [0.04–0.16] (see forest plot, Figure 2). A high level of heterogeneity was observed, *Higgins I*^2^ = 81%. It was noted that one study (Niditch et al., 2012) contributed substantially to the overall level of heterogeneity. Omitting this study, marginally lowered the overall estimate, narrowed the CIs and reduced heterogeneity somewhat, *r* = 0.08, 95% CI: [0.03–0.14], *I*^2^ = 75%. Results of the QEM returned a slightly smaller estimate of the correlation than the REM, *r* = 0.08, 95% CI: [0.01–0.14]. Removing the study that included a small number of participants over the age of 18 made little difference to the estimate of the effect, *r* = 0.09, 95% CI: [0.03–0.15], *I*^2^ = 81%.

#### Subgroup analysis

To understand the relationship between the findings and the inclusion or exclusion of children with ID, subgroup analysis was performed (Figure 2). In all, 15 papers included children with ID, 11 did not include children with ID and for 5 papers it was unclear.

Overall, there was a significant difference between the subgroups, *Q*(2) = 15.87, *p* < 0.001. A clear effect was observed in those studies that included children with ID, *r* = 0.18, 95% CI: [0.11–0.25], *I*^2^ = 82%. There was no significant correlation observed for studies that did not include children with ID, *r* = 0.001, 95% CI: [−0.11 −0.12], *I*^2^ = 58%, and no significant correlation observed for studies that did not report whether children with ID were included, *r* = −0.02, 95% CI: [−0.09 to 0.05], *I*^2^ = 47%.

##### Meta-analysis 2: group-level data

**Figure 3.**
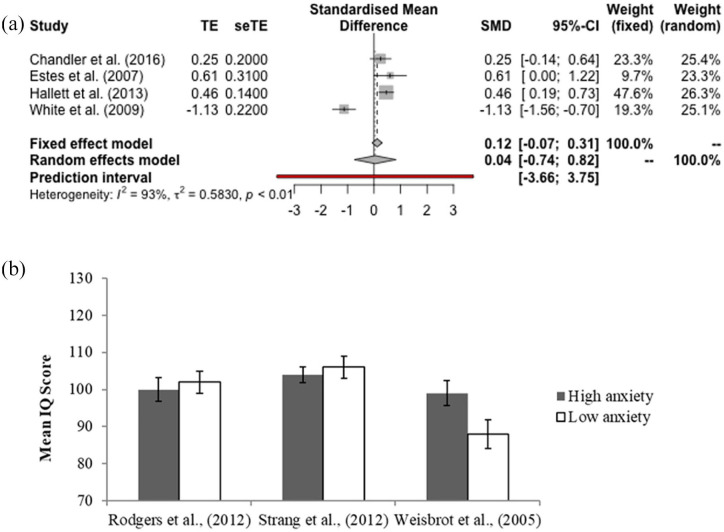
Data from studies with group designs. Panel a presents a forest plot of the random effects model of meta-analysis 2 – on group differences in anxiety between autistic children with and without intellectual disability. Panel b presents the data from studies comparing IQ in high and low anxiety autistic children.

Four papers included group-level comparisons split by IQ, including data from 338 participants with ID and 423 participants without ID. A REM was calculated using the restricted maximum likelihood estimate. The REM indicated that there was not a significant difference between anxiety scores for children with and without ID; *standardised mean difference* (SMD) = 0.04, 95% CI: [−0.74 to 0.82] (see Figure 3, panel a). A high level of heterogeneity was observed, *I*^2^ = 93%. One study (White & Roberson-Nay, 2009) contributed substantially to the overall level of heterogeneity. Omitting this study substantially changed the estimate, returned a significant difference between groups, narrowed the CI and reduced heterogeneity to excellent levels, *SMD* = 0.42, 95% CI: [0.21–0.63], *I*^2^ = 0%. These data supported a difference, such that those with ID scored lower on anxiety measures. Results of the QEM returned a larger estimate of the effect than the REM, but a non-significant difference. *SMD* = 0.16, 95% CI: [−0.63 to 0.96].

### Other analyses

In order to gain clarity on the literature base as a whole, a narrative review is included of papers which were not meta-analysed. Four papers used a median split on anxiety data and compared mean IQ scores between groups, although one study did not report mean IQ scores for high and low anxiety groups (Mazefsky et al., 2011) (Figure 3, panel b). Of these, three studies found no significant difference in IQ scores between high and low anxiety groups, while one (Weisbrot et al., 2005) did not conduct statistical analysis. These studies showed common methodological difficulties, with none employing anxiety measures validated for autistic children with an ID.

A total of 14 papers used other means to look at anxiety scores across lower and higher IQ samples. Of these 14 papers, 6 found a significant result which suggested higher IQ was linked to higher levels of anxiety in autistic children. In total, these six papers included 4760 autistic children. Most of these six papers used multiple analytic methods to assess the relationship between anxiety and IQ. Four papers included chi-square tests and found significant results, whereby high IQ groups had a significantly higher number of children from high anxiety groups (Dubin et al., 2015; Gotham et al., 2012; Sukhodolsky et al., 2008; Witwer & Lecavalier, 2010). Three papers calculated odds ratios, which showed that high IQ children were 1.96–2.9 times more likely to be highly anxious than low IQ children (Dubin et al., 2015; Salazar et al., 2015; Sukhodolsky et al., 2008). Four papers included regression analyses, where IQ scores were significant contributors in predicting anxiety group membership (Dubin et al., 2015; Gotham et al., 2012; Salazar et al., 2015; Sukhodolsky et al., 2008). One study did not conduct any statistical analysis, but reported that 80% of their high IQ group met a clinical cut-off for anxiety, compared to 67% of their low IQ group (Mayes, Calhoun, Murray, Ahuja, et al., 2011). These results must be interpreted with caution as most of these studies used measures of anxiety that have not been clearly validated for their population.

Eight papers (including 2746 autistic children) did not report a significant relationship between anxiety and ID in autism. Of the analyses conducted, five used some form of regression and found no significant result, where IQ scores were not significant predictors of anxiety group membership (Gardiner & Iarocci, 2018; Hollocks et al., 2014b; Kerns et al., 2014; Rosenberg et al., 2011; Simonoff et al., 2008). Two used chi-square tests and found non-significant results, whereby high IQ groups did not have a significantly higher number of children from high anxiety groups (Factor et al., 2017; Kerns et al., 2020). One study conducted a path analysis where children with higher IQs did not have significantly higher anxiety scores (Wijnhoven et al., 2018).

In addition to finding a non-significant relationship between IQ and anxiety in autistic children when using a regression analysis (as discussed above), Rosenberg et al. (2011) also conducted a chi-square test and found that autistic children with comorbid ID were more likely to be anxious than autistic children without ID. The ID group had a significantly higher proportion of children with anxiety disorders than the non-ID group.

## Discussion

Our systematic review included 49 studies. Meta-analysis of results from studies using correlational approaches showed that autistic children with higher IQ scored higher on measures of anxiety – though a relatively small proportion of variance in anxiety was explained. This effect was strongest in those studies that included children with ID and was not significant in those that did not. Comparing anxiety levels in groups of autistic children with lower and higher IQs has been a less frequent approach. Here, only four studies were available, and data were less clear. Three statistically homogeneous studies suggested a statistically significant difference, equivalent to a small-medium effect size, such that those autistic children without comorbid ID experienced higher levels of anxiety. One study (White & Roberson-Nay, 2009) contributed substantially to the overall level of heterogeneity. When removed, the effect estimate changed substantially, returning a significant difference between groups. The CIs narrowed, and heterogeneity was reduced to excellent levels. This study had substantially smaller sample size than the other three studies and scored poorly on the quality framework. Analyses omitting this study are likely a truer reflection of the effect estimate. Four studies compared IQ scores in high and low anxiety groups, and of these, three found no clear differences. Of the 14 papers taking alternative approaches, many using multiple methods of analysis, 6 found significant results consistent with higher levels of anxiety being associated with higher IQ (*n* = 4760), but 7 studies found no relationship between IQ and anxiety (*n* = 2746), and a particularly large study (Rosenberg et al., 2011) found higher rates of anxiety diagnosis in autistic children *with* comorbid ID.

### How and why are anxiety and IQ related in autism?

The findings of this review broadly support the hypothesis that autistic children with a higher IQ have higher levels of anxiety than autistic children with a lower IQ. For studies that included children with IQs across the full range, there was a small, but clear, positive correlation. Similarly, groups of autistic children with ID showed lower anxiety than those without. For studies that only included children with IQs in the normal range or above, there was not consistent evidence of a correlation between IQ and anxiety. The current evidence affords a number of plausible alternatives as to why there was a different pattern across these studies. One possibility is that the relationship between IQ and anxiety exists linearly across the full range of IQs. Testing across a narrower IQ range would predict smaller differences in anxiety and thus require greater statistical power to identify the effect. In support of this, a previous meta-analysis (van Steensel & Heeman, 2017) looked solely at children with IQ in the normal range and identified that the biggest differences between anxiety of autistic children and their peers were in those with highest IQ. A second possibility is that the correlation is underpinned by a non-linear relationship, such that large differences between those with the lowest IQs and those with IQ in the normal range are responsible for producing a small effect overall. The group-level data go some way to support the possibility of this. Finally, there may be methodological differences, such that those studies which excluded those with ID may have shared common methodological constraints. The current literature does not allow for delineation of these alternatives and further research, perhaps including re-analysis of existing data sets, is important.

The papers reviewed proposed a range of possible mechanisms for why IQ and anxiety are related in autistic children. Some authors propose that this link relates directly to cognitive abilities associated with high IQ. High IQ allows greater abstract thinking and planning, which may lead to greater pre-emptive worries and associated anxiety (Kerns & Kendall, 2012). Furthermore, children with a higher IQ may be more capable of higher order functions, which might facilitate worries about the past, future or self-efficacy – which may perpetuate anxiety (Salazar et al., 2015). This pattern is, however, unclear in children without autism (with contradicting evidence available, Karpinski et al., 2018; Martin et al., 2010; Penney et al., 2015). Alternative explanations suggest that higher IQ may interact with social and functional experiences and expectations of autistic children. Higher IQ may precipitate greater exposure to a wider range of environments and social situations – such as mainstream school. These provide exposure to more anxiety provoking situations (Salazar et al., 2015). Alternatively, autistic children with higher IQs may have a better ability to recognise the discrepancy between their social skills and their peers, which might precipitate anxiety (Acker et al., 2018).

However, degree of sensitivity of measurement may account for these findings, as opposed to differences in experience. Assessing anxiety in autistic children is challenging as many behavioural signs of anxiety often overlap with autism symptomatology (Wood & Gadow, 2010). Doing so with those with lower IQ adds additional difficulty (Bailey & Andrews, 2003). This is in part due to overlap in behavioural symptoms of anxiety with undiagnosed health conditions, which are common in ID. For example, gastrointestinal disorders occur in up to 73% of autistic children with comorbid ID and often lead to sleep difficulties, aggression and self-injurious behaviour (Mannion & Leader, 2016). Autistic children with higher IQ are likely better at expressing their worries than autistic children with comorbid ID (Salazar et al., 2015). Measures of anxiety are rarely adapted for use in autistic children with ID. These measures typically rely on parent reports of anxiety, which relies on a child’s ability to label and express their emotions to their caregiver. Alternatively, these measures may require parents to notice behaviours associated with anxiety, which are often qualitatively different in children with ID (Bailey & Andrews, 2003).

Discriminating between these broad alternatives should be a priority for research in the area, as it has key clinical implications. If anxiety is truly increased in autistic children with high IQ, further work is needed to assess its impact on quality of life and functioning. While autistic children with higher IQs are more likely to be clinically anxious, these children are also likely to have better coping mechanisms for anxiety. Higher levels of anxiety and concomitant impact on quality of life would support directing more resources to anxiety in autistic children with higher IQ. If the differences identified reflect measurement sensitivity, the exact opposite is true; extra resources are needed for those with lower IQ to improve assessment. Either way, current policy makers and service providers should exercise restraint. Methodological limitations need to be resolved and the current estimate suggests a relatively small degree of variance in anxiety is explained by IQ in autistic children.

### Comparison to other reviews

This is the first meta-analytic review of this research question. It provides an interesting point of comparison to a recent review in autistic adults. Hollocks et al. (2019) meta-analysed prevalence rates of anxiety and depression in autistic adults, and if having comorbid ID had any impact on this. They found that studies including autistic adults and comorbid ID had lower prevalence estimates of anxiety than studies which contained autistic adults alone, though these results did not reach significance. These results are broadly in line with those we find in autistic children. A second meta-analysis (van Steensel & Heeman, 2017) investigated whether rates of anxiety were higher in autistic children than typically developing children. Their results showed that anxiety disorders were more prevalent in autistic children than typically developing children, and that this effect was moderated by IQ. That is, the higher the IQ of the autistic children, the larger the discrepancy between anxiety levels of the autistic children compared to the typically developing children. van Steensel and Heeman’s (2017) results support those found in this review and suggest that the mechanism relating IQ and anxiety may be specific to autistic children. Notably, van Steensel and Heeman (2017) excluded children with ID, meaning less is known about the mechanism in this group (~50% of autistic children; Charman et al., 2011).

### Limitations

The literature proved sufficient for the completion of two methodologically sound meta-analyses, with additional papers providing narrative reports. It remains limited in important ways. One limitation of the present review is the heterogeneity of the literature (*I*^2^ = 81% and 77%). Notably, in each case, this was substantially reduced by removing a single study. For the meta-analysis of correlations, it was unclear why this study (Niditch et al., 2012) returned such differences, so they may reflect something specific to the population used. For the group-level meta-analysis, this was likely explained by the small sample size and methodological quality (White & Roberson-Nay, 2009).

Different papers used different methods to assess autism and IQ, but the largest methodological variation was in measures of anxiety, as there is no gold standard means of assessing anxiety in autistic children with comorbid ID. It is therefore unclear if anxiety measures across papers are necessarily measuring the same construct.

The results of any meta-analysis reflect the ability to gather and extract data from search results returned and decisions made in analytical strategy. Here, given the relatively small and heterogeneous nature of the literature, we chose to include papers where possible. One study was included that had participants with an average age <18 years, but a small number of participants age 18–25 (Syriopoulou-Delli et al., 2019). Omitting this paper made little difference to the overall estimate. Three sets of authors were unable to provide data for a paper returned by the search, and these papers all reported a non-significant correlation (Johnson et al., 2015; Mayes & Calhoun, 2004; Ozsivadjian et al., 2014). Therefore, it is possible that results are a marginal over-exaggeration of true effect sizes.

Meta-analytic processes are often limited by the original analytic decisions made by authors of the papers returned. All 31 of the papers that included correlations investigated a linear relationship between IQ and anxiety. Our meta-analysis raises an important question as to whether this was appropriate, given that those studies excluding those with the lowest IQs did not identify an effect. Synthesising the data in this way was vital, given prevailing approach in the literature. Our data make clear that future consideration of other analytical approaches is needed.

### Future research and clinical implications

Alongside establishing more clearly the nature of the relationship between IQ and anxiety in autistic children, future research is needed to improve our methods of assessing and treating anxiety in this group. In particular, future research should focus on the development of measures for anxiety in autistic children, which are suitable for those with comorbid ID. Results of the quality scoring indicated that this was an area of particular weakness of papers included in this review, with methods chosen often being unsuitable for use in autism and ID. Having a gold standard method of measurement of anxiety will not only make research easier to compare and draw valid conclusions from, but would also be a useful clinical tool to aid diagnosis of anxiety in these groups. Psychometric properties of the recently developed Anxiety Scale for Children – ASD (Rodgers et al., 2016) and the Parent-Rated Anxiety Scale for ASD (Scahill et al., 2019) are promising, but further research is needed to validate these measures in larger populations. Finding the mechanism by which IQ influences anxiety in autistic children is critical, as only then can treatments be offered which are tailored to specific needs including IQ level. It is clear that anxiety disorder interventions can be easily modified and effective for autistic children (van Steensel & Bogels, 2015), but treatment response in autistic children with an ID is rarely investigated. Anxiety levels are high across autistic children, whether or not they have a comorbid ID (Mayes, Calhoun, Murray, Ahuja, et al., 2011). Interventions for anxiety in autistic children should be established which are appropriate for children with comorbid ID, as they have been overlooked in the literature to date. Most work into treatments for anxiety in autism, such as modified CBT, has focused on children with higher intellectual abilities (van Steensel & Bogels, 2015).

## Conclusion

The existing literature shows a significant relationship between high IQ and high levels of anxiety in autistic children. Existing methods for measuring anxiety in autistic groups with comorbid ID are poor. Few measures exist which are appropriately modified for this group and often rely on verbal labelling of complex emotional states. Future work is needed to confirm whether the relationship between IQ and anxiety in autistic children is primarily driven by experience or measurement.

## Supplemental Material

sj-pdf-1-aut-10.1177_1362361320953253 – Supplemental material for Anxiety and intellectual functioning in autistic children: A systematic review and meta-analysis Click here for additional data file. Supplemental material, sj-pdf-1-aut-10.1177_1362361320953253 for Anxiety and intellectual functioning in autistic children: A systematic review and meta-analysis by Jessica E Mingins, Joanne Tarver, Jane Waite, Chris Jones and Andrew DR Surtees in Autism

## Notes

1. Throughout this review, the term children is used to refer to groups of children and adolescents under age 18.
