# Supplementary material for: Anxiety and intellectual functioning in autistic children: A systematic review and meta-analysis
Source: Autism. 2020 Nov 16;25(1):18–32. doi: 10.1177/1362361320953253 (PMC8162138; doi:10.1177/1362361320953253)
Supplement: sj-pdf-1-aut-10.1177_1362361320953253 – Supplemental material for Anxiety and intellectual functioning in autistic children: A systematic review and meta-analysis [file sj-pdf-1-aut-10.1177_1362361320953253.pdf]

Supplementary Table 1. Full descriptions of each paper included in this review.

Author (year)	N	Age range (mean)	% Male	IQ Range (mean)	Recruitment	Autism	Anxiety	IQ	Respondent	Results	Quality Score (/3) Sample	Autism	IQ	Anxiety	Statistics	Total
**Correlations between anxiety and IQ measures (Meta-analysis 1)**																
Bitsika & Sharpley, (2018)	90	6-12 (8.8)	100	70+ (95.93)	Multiple support groups (Australia)	Existing clinical diagnosis	CASI-Anxiety	WASI, WISC	Self	No significant correlation	2	2	3	3	3	2.6
Chandler et al., (2016)	273	4-8 (6.8)	-	19+ (72.7)	Multiple clinics (UK)	Diagnosis confirmed by clinical consensus	DBC-P	WISC, WPPSI, MSEL	Parent	No significant correlation	2	2	3	2	3	2.4
Conner, Maddox, & White (2013)	30	12-17 (-)	-	70+ (-)	Previous study (USA)	Existing clinical diagnosis	CASI-20	WASI	Parent	No significant correlation	1	2	2	2	3	2
Dukevot et al., (2018)	123	2-10 (6.7)	81.5	50-141 (96.4)	Previous Study (Netherlands)	Best estimate diagnosis by clinician	CBCL	WISC-III, WPPSI-III, SON-R, BSID, WASI	Parent	No significant correlation	2	2	2	1	3	2
Eussen et al., (2013)	134	6-13 (9.22)	88	48-128 (91.4)	Outpatient centre (Netherlands)	Diagnosis confirmed by clinical consensus	CBCL	WISC	Parent	No significant correlation	1	2	3	1	3	2
Factor et al., (2017)	44	3-17 (7.25)	82.5	- (90.98)	Emails, phone calls, posters (USA)	Diagnosis by clinical consensus	CBCL	MSEL, WPPSI-IV, WASI-II, WISC-V	Parent	No significant correlation	2	3	3	1	3	2.4
Gadow et al., (2008)	238	6-12 (8.61)	86	70+ (98)	Previous study (USA)	Existing clinical diagnosis	CSI	School reports	Parent	Significant positive correlation	2	2	1	3	3	2.2
Gadow et al., (2016)	214	6-18 (10.4)	81	- (-)	University hospital (USA)	Diagnosis confirmed by clinical consensus	CASI-Anxiety	School reports	Parent	No significant correlation	1	2	1	2	3	1.8
Hallett et al., (2013a)	107	10-15 (13.5)	85	50+ (88.07)	Previous study (UK)	Diagnosis confirmed by	RCADS	WASI, BPVS, RM	Parent	No significant correlation	2	3	3	1	3	2.4

Author (year)	N	Age range (mean)	% Male	IQ Range (mean)	Recruitment	Autism	Anxiety	IQ	Respondent	Results	Quality Score (/3)					
											Sample	Autism	IQ	Anxiety	Statistics	Total
						clinical consensus										
Hill et al., (2014)	102	6-13 (-)	82	- (79.55)	Archival hospital #data (USA)	Existing clinical diagnosis	BASC	WPPSI, WISC	Parent	Significant positive correlation	1	2	2	2	3	2
Hollocks et al., (2014a)	90	14-16 (15.5)	91.1	50-119 (84.5)	Project cohort (UK)	Diagnosis confirmed by multiple measures	SDQ, PONS	WASI	Parent	Significant negative correlation	2	2	3	1	3	2.2
Hollocks et al. (2016)	55	10-16 (12.8 [ASDAnx]/ 13.0 [ASD])	100	76-138 (99.7 [ASDAnx]/103 [ASD])	Multiple clinics (UK)	Existing clinical diagnosis	SCAS-P	WASI	Parent	No significant correlation	2	2	3	2	3	2.4
Johnston and Iarocci, (2017)	67	6-14 (9.82)	85	74-139 (102.59)	Previous study (Canada)	Existing clinical diagnosis	BASC	SB, WASI	Parent	No significant correlation	1	2	3	2	3	2.2
Kaat & Lecavalier, (2015)	46	8-16 (12.4)	83	56-127 (90.7)	Multiple schools, clinics, research registry (USA)	Diagnosis confirmed by one measure	MASC	WISC-IV	Parent	No significant correlation	2	2	3	1	3	2.2
Kelly et al., (2008)	322	6-16 (10.9)	-	-	Two specialist clinics (Australia)	Existing clinical diagnosis or elevated ASASD score	SDQ	Unspecified	Parent	No significant correlation	2	1	0	2	3	1.6
Kerns et al., (2020)	62	9-13 (11.5)	80	25-170 (77.39)	Database, advertising at schools & pediatric clinics (USA)	Diagnosis confirmed by multiple measures	MASC	DAS-II	Parent	Significant positive correlation	2	2	3	2	3	2.4
Mayes et al., (2011b)	627	1-17 (6.6)	86	16-146 (88)	Diagnostic clinic (USA)	Diagnosis confirmed by clinical consensus	Pediatric behavior scale	WISC, WPPSI, SB, Bayley Mental Scale	Parent	Significant positive correlation	1	2	2	1	3	1.8
Mazefsky, Kao & Oswald (2011)	38	10-17 (12)	82	71-144 (105)	One clinic (USA)	Diagnosis confirmed by multiple measures	RCMAS, ACI-PL	WASI	Parent	Significant negative correlation	1	2	3	3	3	2.4

											Quality Score (/3)					
**Author (year)**	**N**	**Age range (mean)**	**% Male**	**IQ Range (mean)**	**Recruitment**	**Autism**	**Anxiety**	**IQ**	**Respondent**	**Results**	**Sample**	**Autism**	**IQ**	**Anxiety**	**Statistics**	**Total**
Mazurek et al., (2014)	225	2-17 (7.9)	86.7	- (82.5)	Treatment network (USA)	Diagnosis confirmed by one measure	CBCL	SB-5, WPPSI-III, MSEL, WASI	Parent	No significant correlation	2	2	3	1	3	2.2
Mazurek & Petroski (2015)	1347	2-17 (7.9)	84.8	- (90.56 [2-5yrs] /85.56 [6+yrs])	Online database (USA & Canada)	Diagnosis confirmed by clinical consensus	CBCL	SB-5, WISC-IV, WPPSI-III, WASI, DAS-II, MSEL	Parent	No significant correlation	2	2	2	1	3	2
Niditch et al., (2012)	231	2-9 (5.0)	84	- (64.2)	Archival hospital data (USA)	Diagnosis confirmed by clinical consensus	BASC-Anxiety	MSEL, WPPSI, WISC, Leiter-R, WNV	Parent	Significant positive correlation	1	2	2	2	3	2
Oswald et al., (2016)	32	12-17 (14.86)	56	75+ (110.16)	Previous study databases, advertising (USA)	Existing clinical diagnosis	RCADS-P	KBIT-2	Parent	No significant correlation	2	2	3	1	3	2.2
Pearson et al., (2007)	26	4-17 (9.5 [ASD] /10.5 [PDD])	96.2 [ASD]/ 76 [PDD]	44-134 (75 [ASD]/ 94 [PDD])	Retrospective clinical cases & research (USA)	Diagnosis confirmed by clinical consensus	PIC-R	SB-5, WISC-III, WPPSI-R	Parent	No significant correlation	1	2	2	3	3	2.2
Rodas, Eisenhower, & Blacher (2017)	159	4-7 (5.5)	82	- (93.5)	Previous study (USA)	Diagnosis confirmed by one measure	CBCL	WPPSI-III	Parent	No significant correlation	2	2	2	1	3	2
Rosen & Lerner, (2017)	51	7-17(12.15)	74.5	71-133 (102.82)	Previous study (USA)	One measure (ADOS-2)	MASC-2	KBIT-2	Parent	No significant correlation	1	2	2	2	3	2
Russell, Frost, & Ingersoll, (2019)	2093	5-18 (10.3)	86.6	7-167 (81.55)	Multiple adverts-parent grounds, TV, radio, & more (USA)	Diagnosis confirmed by multiple measures	CBCL	DAS-II, MSEL, WISC-IV, WASI	Parent	Significant positive correlation	2	2	2	1	3	2
South et al. (2010)	24	9-21 (14.03)	91.6	87-132 (109.71)	- (USA)	Diagnosis confirmed by one measure	SCARED	WASI	Parent	Significant negative correlation	0	2	3	2	3	2
Syriopoulou-Delli et al., (2018)	291	4-25 (10)	73.5	50-150 (91.33)	Multiple specialist schools (Greece)	Existing clinical diagnosis	SAS	Unspecified	Teacher	Significant positive correlation	2	1	0	1	3	1.4
Vasa et al., (2013)	1316 (557 [2-	2-17 (3.5 [2-5 yrs]/	83 (2-5 yrs)/	73.6 (2-5 yrs) /	14 outpatient centres	Diagnosis confirmed by	CBCL	SB-V, MSEL	Parent	Significant positive correlation	2	2	3	1	3	2.2

											Quality Score (/3)					
**Author (year)**	**N**	**Age range (mean)**	**% Male**	**IQ Range (mean)**	**Recruitment**	**Autism**	**Anxiety**	**IQ**	**Respondent**	**Results**	**Sample**	**Autism**	**IQ**	**Anxiety**	**Statistics**	**Total**
	5 yrs]/ 346 [6-11 yrs]/ 115 [12-17 yrs])	7.8 [6-11 yrs] /13.9 [12-17 yrs])	85 (6-11 yrs)/ 85 (12-17 yrs)	80.8 (6-11 yrs)/ 76.8 (12-17 yrs)	(USA and Canada)	clinical consensus										
White, Schry, & Maddox, (2011)	30	12-17 (14.58)	76.6	70+ (97.07)	Previous study (USA)	Existing clinical diagnosis	MASC-2, CASI-Anx	WASI	Parent	No significant correlation	1	2	2	2	3	2
Wigham et al., (2015)	53	8-16 (12.49)	88.68	70+ (106.2)	Multiple databases (UK)	Existing clinical diagnosis	SCAS-P	WASI	Parent	No significant correlation	2	2	3	2	3	2.4
**Group level data split by high vs low IQ scores (meta-analysis 2)**																
Chandler et al., (2016)	226	4-8 (6.8)	-	19+ (72.7)	Multiple clinics (UK)	Diagnosis confirmed by clinical consensus	DBC-P	WISC, WPPSI, MSEL	Parent	Autistic high IQ significantly more anxious than low IQ	2	2	3	2	3	2.4
Estes et al., (2007)	57	6-9 (6.1)	82	-	Previous study, multiple schools, clinics and hospitals (USA)	Existing clinical diagnosis	CBCL	DAS	Parent	Autistic high IQ significantly more anxious than autistic low IQ	2	2	3	1	3	2.2
Hallett et al., (2013b)	392	4-17 (8.47)	85	-	Previous study (USA)	Existing clinical diagnosis	CASI-Anxiety	WISC, WPPSI, MSEL, SIT, Leiter-R	Parent	Autistic high IQ significantly more anxious than autistic low IQ	1	1	2	1	3	2.2
White and Roberson-Nay, (2009)	17	7-14 (12.08)	90	- (92)	Outpatient clinic (USA)	Existing clinical diagnosis	MASC	Unspecified	Self	No significant difference between high and low IQ groups	1	2	0	1	3	1.4
**Group level data split by high vs low anxiety (not meta-analysed)**																
Rodgers et al., (2012)	67	8-16 (11.2)	87	-	Two previous studies (UK)	Existing clinical diagnosis	SCAS-P	WASI	Parent	No significant difference between high and low IQ groups	2	2	3	2	3	2.4
Strang et al., (2012)	95	6-18 (11.67)	86	71-144 (105)	Diagnostic clinic (USA)	Existing clinical diagnosis	CBCL	WISC, WASI	Parent	No significant difference between high and low IQ groups	1	2	3	2	3	2.2

Author (year)	N	Age range (mean)	% Male	IQ Range (mean)	Recruitment	Autism	Anxiety	IQ	Respondent	Results	Quality Score (/3)					
											Sample	Autism	IQ	Anxiety	Statistics	Total
Weisbrot et al., (2005)	75	3-12 (-)	-	-	Two outpatient clinics (USA)	Existing clinical diagnosis	CSI-4, ECI-4	Unspecified	Parent	High anxiety had higher IQ than low anxiety	2	1	0	2	3	1.4
**Other analyses (not meta-analysed)**																
Dubin et al., (2015)	2662	4-17 (8.82)	86.4	- (-)	Online database (USA)	Best estimate diagnosis by clinician	CBCL	DAS, WISC, MSEL, WASI	Parent	Significant positive relationship (Chi-squared, regression)	2	2	2	1	3	2
Gardiner & Iarocci (2018)	59	5-13 (10.1)	86.4	87-144 (107.47)	Archival data (Canada)	Diagnosis confirmed by multiple measures	BASC-C	WASI-II	Parent	No significant relationship (Multiple regression)	0	2	2	1	3	1.6
Gotham et al., (2012)	1429	5-18 (10.2)	86	5-167 (80)	Online database (USA)	Diagnosis confirmed by multiple measures	CBCL	SB-5, WISC-IV, WPPSI-III, WASI, DAS-II, MSEL	Parent	Significant positive relationship (Chi-squared, regression)	2	2	2	1	3	2
Hollocks et al., (2014b)	52	10-16 (12.9)	100	76-138 (103)	Multiple clinics (UK)	Existing clinical diagnosis	SCAS-P, SCAS-C	WASI	Parent and self combined	No significant relationship (Linear regression)	2	2	3	2	3	2.4
Kerns et al., (2014)	59	7-17 (10.48)	78	67-158 (104.69)	Database of families, online registry (USA)	Diagnosis confirmed by multiple measures	ADIS-C/P	DAS-II, WISC-IV	Parent and self combined	No significant relationship (regression)	2	2	3	2	3	2.4
Mayes et al., (2011a)	350	6-16 (8.0)	-	- (-)	Diagnostic clinic (USA)	Existing clinical diagnosis	Pediatric behavior scale	WISC, WASI	Parent	Significant positive relationship (Comparing percentages)	1	2	2	1	3	1.8
Rosenberg et al., (2011)	4343 (X^2) 2219 (regression)	5-18 (8.9)	83.47	- (-)	National online database (USA)	Existing clinical diagnosis	Parent report of anxiety disorder	Parent report of ID status	Parent	Significant negative relationship (Chi-squared) No significant relationship (Regression)	2	0	0	0	3	1
Salazar et al., (2015)	101	4-9 (6.7)	56	19-120 (72.8)	Multiple support groups and primary care services (UK)	Diagnosis confirmed by clinical consensus	PAPA	MSEL, WPPSI, WISC	Parent	Significant positive relationship (Regression)	2	2	3	1	3	2.2
Simonoff et al., (2008)	112	10-14 (11.5)	87.5	19-124 (72.7)	Project cohort (UK)	Diagnosis confirmed by	CAPA	WISC, RM	Parent	No significant relationship (Regression)	2	3	3	2	3	2.6

Author (year)	N	Age range (mean)	% Male	IQ Range (mean)	Recruitment	Autism	Anxiety	IQ	Respondent	Results	Quality Score (/3) Sample	Autism	IQ	Anxiety	Statistics	Total
						clinical consensus										
Sukhodolsky et al., (2008)	154	5-17 (8.2)	84	19+ (-)	Previous study (USA)	Existing clinical diagnosis	CASI-Anxiety	WISC, WPPSI, MSEL, SIT, Leiter-R	Parent	Significant positive relationship (Chi-squared, regression)	2	2	2	2	3	2.2
Winjhoven et al., (2018)	141	8-15 (11.25)	77.9	- (104.87)	Two mental health institutes, one special education school (Netherlands)	Existing clinical diagnosis	SCAS-P	WISC, WPPSI, RAKIT, SON-R	Parent	No significant relationship	2	2	2	2	3	2.2
Witwer and Lecavalier, (2010)	58	6-17 (11.2)	82	42-150 (68.4)	Multiple clinics, support groups, and mailing lists (USA)	Existing clinical diagnosis	P-ChIPS	SB	Parent	Significant positive relationship (Chi-squared)	2	2	3	1	3	2.2

ACI-PL, Autism Comorbidity Interview- Present and Lifetime Version; **ADIS,** Anxiety disorder interview schedule; **ADOS**, Autism Diagnostic Observation Schedule; **ASASD**, Australian Scale for Autism Spectrum Disorder; **BASC**, Behaviour Assessment System for Children; **BPVS**, British Picture Vocabulary Scale; **BSID**, Bayley Infant Scale of Development; **CAPA**, Child and Adolescent Psychiatric Assessment; **CASI**, Child and Adolescent Symptom Inventory; **CBCL**, Child Behaviour Checklist; **CSI-4**, Child Symptom Inventory-4; **DAS**, Differential Ability Scale; **DBC**, Developmental Behaviour Checklist; **ESI-4**, Early Symptom Inventory-4; **KBIT**, Kaufman Brief Intelligence Test; **KID-SCID**, Structured Clinical Interview for DSM-IV Psychiatric Diagnoses; **Leiter-R**, Leiter International Performance Scale; **MASC**, Multidimensional Anxiety Scale for Children; **MSEL**, Mullen Scales of Early Learning; **PAPA**, Preschool Age Psychiatric Assessment; **P-ChIPS**, Children's Interview for Psychiatric Symptoms- Parent; **PIC-R**, Personality Inventory for Children; **PONS**, Profile of Neuropsychiatric Symptoms; **RAKIT**, Revisie Amsterdamse Kinder Intelligentietest; **RCADS**, Revised Children's Anxiety and Depression Scale; **RCMAS**, Revised Children's Manifest Anxiety Scale; **RM**, Raven's Matrices; **SB**, Stanford-Binet; **SCARED**, Screen for Child Anxiety Related Disorders; **SCAS-P/C**, Spence Children's Anxiety Scale- Parent/Child; **SDQ**, Strengths and Difficulties Questionnaire; **SIT**, Slosson Intelligence Test; **SON-R**, Snijders-Oomen Nonverbal IQ Test; **WASI**, Wechsler Abbreviated Scale of Intelligence; **WISC**, Wechsler Intelligence Scale for Children; **WNV**, Wechsler Nonverbal Measure of Ability; **WPPSI**, Wechsler Preschool and Primary Scale of Intelligence.

Note. Chandler et al., (2016) appears in both meta-analysis 1 and meta-analysis 2. Where papers have two or more reported means for age or IQ score, these studies split participants into groups based on age or diagnosis and means for all groups are reported in this table.
